# Real-time vascular monitoring of PDLs and IPL for vascular treatment: effects of fluence and pulse duration on a rabbit ear model

**DOI:** 10.3389/fmed.2026.1917018

**Published:** 2026-08-28

**Authors:** Tun Tun Aung, Yusang Hu, Xiaohan Liu, Manmei Long, Wanyi Wang, Khin Maung Htwe, Soe Lei May, Yuhao Sun, Yixin Zhang, Zheng Zhang

**Affiliations:** 1 Department of Plastic and Reconstructive Surgery, Shanghai Ninth People’s Hospital, School of Medicine, Shanghai Jiao Tong University, Shanghai, China; 2 Department of Pathology, Shanghai Ninth People’s Hospital, School of Medicine, Shanghai Jiao Tong University, Shanghai, China; 3 Department of Laser and Aesthetic Medicine, Shanghai Ninth People’s Hospital, Shanghai JiaoTong University School of Medicine, Shanghai, China; 4 School of Medicine, Shanghai Jiao Tong University, Shanghai, China; 5 College of Stomatology, Shanghai Jiao Tong University, Shanghai, China

**Keywords:** animal model, dermoscopy, intense pulsed light, laser speckle contrast imaging, laser treatment, pulsed dye laser, rabbit, vascular monitoring

## Abstract

**Background:**

Pulsed dye lasers (PDLs) and intense pulsed light (IPL) are commonly used for treating cutaneous vascular diseases but lack standardized and reproducible treatment protocols. Reliable vascular models are needed to visually assess posttreatment responses.

**Methods:**

Sixteen male rabbits were divided into two groups, with each ear subdivided into three treatment regions. PDL treatment was applied with pulse durations of 0.45 ms (fluences: 4, 5.5, and 8 J/cm^2^) and 1.5 ms (fluences: 5.5, 8, and 13 J/cm^2^). IPL was applied in single-pulse (10, 13, and 15 J/cm^2^) and double-pulse (10, 13, 17, and 20 J/cm^2^) modes. Vascular morphology and blood flow were evaluated using photographs and laser speckle contrast imaging (LSCI) before treatment, immediately after treatment, on days 1, 3, 5, 7, and 14. Histopathological analysis was performed 24 hours posttreatment. Statistical analysis was conducted with two-way ANOVA and Tukey's test, with *P* < 0.05 considered to indicate statistical significance.

**Results:**

The dorsal ear vessels of rabbits consisted of longitudinally arranged main blood vessels (MBVs) and fan-shaped branch blood vessels (BBVs), with diameters ranging from 150–300 μm and 90–120 μm, respectively. In the PDL treatment, effective vessel closure with limited peripheral tissue damage was achieved using 0.45 ms at 5.5 J/cm^2^ for BBVs, whereas 1.5 ms at 8.0 J/cm^2^ was associated with qualitative evidence of more sustained MBV closure. In the IPL treatment, a single pulse at 13.0 J/cm^2^ effectively closed the branch vessels, whereas the double-pulse mode primarily induced vessel dilation.

**Conclusion:**

The rabbit ear vascular model, combined with dermoscopy, LSCI, and histopathology, provides a reproducible multimodal platform for longitudinal evaluation and systematic parameter screening, offering a reliable preclinical foundation for comparative assessment and protocol refinement prior to clinical translation.

## 1 Introduction

Laser and light-based therapies serve as indispensable modalities in the management of cutaneous vascular lesions, including port-wine stains (PWS) and hemangiomas, primarily due to their minimal invasiveness, rapid recovery, and superior safety profiles (1–3). Pulsed dye lasers (PDLs) and intense pulsed light (IPL) remain the two most prevalent modalities, both of which operate on the basis of the principle of selective photothermolysis. By targeting oxyhemoglobin, PDL and IPL devices convert light energy into thermal energy, inducing localized vascular coagulation and mechanical disruption (4, 5). Specifically, a PDL utilizing monochromatic laser light at either 585 nm or 595 nm is recognized as the clinical gold standard for superficial vessels because of its high selectivity; however, its efficacy is often limited by a shallow penetration depth. In contrast, IPL generates a broad spectrum of noncoherent light (500–1200 nm). When equipped with specialized vascular filters (530–650 nm and 900–1200 nm), IPL facilitates deeper penetration into the dermal vasculature by capturing multiple hemoglobin absorption peaks (6, 7). Furthermore, the large spot size of IPL enhances therapeutic efficacy. These combined advantages allow for the simultaneous management of heterogeneous vascular lesions and associated dyschromia, thereby offering broader clinical utility in complex cases (8, 9).

Although the therapeutic efficacy of PDL and IPL for vascular lesions is well documented, substantial discrepancies in treatment parameters persist throughout the literature. Consequently, parameter selection in clinical practice remains a subject of ongoing controversy, and a standardized protocol has yet to be established (10, 11). This clinical dilemma stems from two primary factors. First, the vast array of adjustable variables, including pulse duration, fluence, and pulse mode, and their complex interactions collectively dictate the final therapeutic outcomes (12, 13). Second, the high degree of interpatient variability in vessel depth, Fitzpatrick skin types and therapeutic objectives necessitate a delicate balance between vascular efficacy and tissue safety (14, 15). Therefore, developing a reliable animal vascular model to systematically evaluate both acute vascular responses and long-term remodeling under various laser parameters is crucial for comparative assessment and preclinical refinement of treatment protocols (16). Furthermore, integrating noninvasive monitoring technologies may provide objective and longitudinal measures of treatment-induced vascular responses, facilitating comparative assessment and preclinical refinement of treatment parameters (17, 18).

In the ongoing effort to refine vascular laser interventions, several animal models have been utilized to explore vessel‒light interactions and systematically evaluate vascular responses to different treatment parameters. Although chick chorioallantoic membrane (CAM) and rat mesentery allow the direct visualization of vascular dynamics, their lack of complete cutaneous histological structure fails to replicate the complex physiological environment, vascular remodeling, and tissue repair processes (19–22). Similarly, although the mouse dorsal skinfold window chamber provides high-resolution microvascular imaging, its invasive nature limits long-term follow-up and increases infection risk (23, 24). These limitations highlight the need for experimental approaches that combine accessible cutaneous vasculature with repeatable, noninvasive, and longitudinal assessment.

The rabbit ear vascular model represents a well-established experimental platform characterized by a clear anatomical architecture and abundant blood supply, and has been extensively utilized in hemodynamic monitoring and various vascular disease investigations (25, 26). Previous studies have primarily employed this model to construct vascular embolism models, for instance, by injecting filler materials into the central ear artery to simulate vascular occlusion and evaluate therapeutic interventions (27, 28). Other studies have utilized ear veins to compare responses to cryotherapy and Nd:YAG laser treatment; however, these observations were confined to morphological changes in the central dorsal veins, without multidimensional assessment of vascular responses or systematic investigation of the relationship between laser parameters and therapeutic efficacy (29). Moreover, these studies predominantly relied on invasive assessment methods, such as implanted window chambers for live microscopy and tissue sectioning, which limit repeated noninvasive and longitudinal monitoring (30). Therefore, integrating standardized noninvasive imaging with complementary histopathological assessment within this established vascular model may provide a reproducible approach for longitudinal characterization of laser-induced vascular responses and systematic parameter screening.

In the present study, we applied the established rabbit ear vascular model to develop a standardized multimodal assessment framework integrating dermoscopy, laser speckle contrast imaging (LSCI), and histopathology. Dermoscopy was used to characterize vascular morphology, LSCI provided quantitative assessment of perfusion changes, and histopathology provided complementary tissue-level assessment. Using this framework, we systematically characterized vascular responses to different PDL and IPL parameters. The study aimed to establish a reproducible multimodal approach for comparative vascular response assessment and parameter screening, thereby providing a useful preclinical framework for the further investigation of vascular laser interventions.

## 2 Materials and methods

### 2.1 Animals

Sixteen adult New Zealand White rabbits (males, 2.2–2.5 kg) were provided by Shanghai Jiagan Laboratory Animal Co., Ltd., (China). All experimental procedures involving animals were conducted in accordance with institutional guidelines for the care and use of laboratory animals and were approved by the Animal Ethics Committee of Shanghai Ninth People's Hospital, Shanghai Jiao Tong University. The rabbits were provided ad libitum access to water and a standard diet during the experimental period. Before the experiments, the hair on the dorsal ear surface was removed using a depilatory cream. During image acquisition, the rabbits were secured in a restraining device and remained conscious without the need for anesthesia.

### 2.2 Experimental design

Rabbits were randomly divided into PDL and IPL groups, with 8 rabbits in each group. In each group, 6 rabbits were used for longitudinal follow-up observation, and 2 were used for histopathological examination. Three circular treatment areas (25 mm in diameter) were designated on each rabbit ear, with a minimum spacing of 15 mm to prevent thermal interference. Within each observation window, PDL or IPL irradiation was delivered across the vascular network. Each region corresponded to a single treatment parameter. For each parameter setting, five independent treatment sites from five different rabbit ears were analyzed (*n* = 5).

The pulse duration and inter-pulse interval were selected based on clinically established treatment parameters and published literature, whereas the fluence levels were determined based on previous studies and preliminary pilot experiments to characterize vascular responses without inducing severe tissue injury (31–34).

For PDL treatment (595 nm, Vbeam, Syneron-Candela), pulse durations of 0.45 ms and 1.5 ms were used with a 7 mm circular spot size. The 0.45 ms group received fluences of 4, 5.5, and 8 J/cm^2^; the 1.5 ms group received 5.5, 8, and 13 J/cm^2^. A single pass was performed on the dorsal ear with 20% spot overlap.

For IPL treatment (M22, Lumenis), a vascular filter (530–650 nm & 900–1200 nm) and a 15 × 8 mm spot size were used. The single-pulse mode (5 ms) delivered fluences of 10, 13, and 15 J/cm^2^; the double-pulse mode (30 ms interval) delivered 10, 13, 17, and 20 J/cm^2^. One to two passes were applied with 20% spot overlap.

### 2.3 Dermoscopy and laser speckle contrast imaging (LSCI)

Dermoscopic images were taken using a handheld portable DE 3100 dermoscope (Shenzhen Ibooloo Optics Co., Ltd., Shenzhen, China) combined with a transillumination technique to evaluate rabbit ear vessel morphology. The dermoscopic lens was positioned on the dorsal ear surface (25-mm field of view), with transillumination from a constant-intensity white light source on the ventral side for high-contrast vascular imaging with minimal surface reflection. Dermoscopic vascular responses were assessed according to predefined criteria, including vasoconstriction, vasodilation, vessel occlusion/disappearance, coagulative change, vessel rupture, and erythrocyte extravasation (definitions in Supplementary Table 1). These morphological features were independently and blindly evaluated by two investigators, and multiple features could be recorded within the same observation field given their concurrent nature.

Real-time blood perfusion was recorded using a laser speckle contrast imaging (LSCI) system, with raw speckle images processed by the built-in software (SIM BFI HR Pro, Wuhan SIM Opto-Technology Co., Ltd., Wuhan, China). At each treatment site, five representative branch blood vessels (BBVs) of similar caliber, clearly identifiable on both dermoscopy and LSCI before treatment, were selected as fixed regions of interest (ROIs) (Figure 2). These same ROIs were tracked consistently at all time points, yielding 25 vascular ROIs per energy setting. To minimize caliber-related variability, only BBVs with baseline diameters of 90–120 μm were included in the quantitative analysis.

Perfusion values (PV), expressed in perfusion units (PU), were automatically generated by the LSCI software. The vascular inhibition rate (IR) was calculated as: IR (%) = [(PV baseline - PV post-intervention)/PV baseline] × 100%.

**Figure 1:**
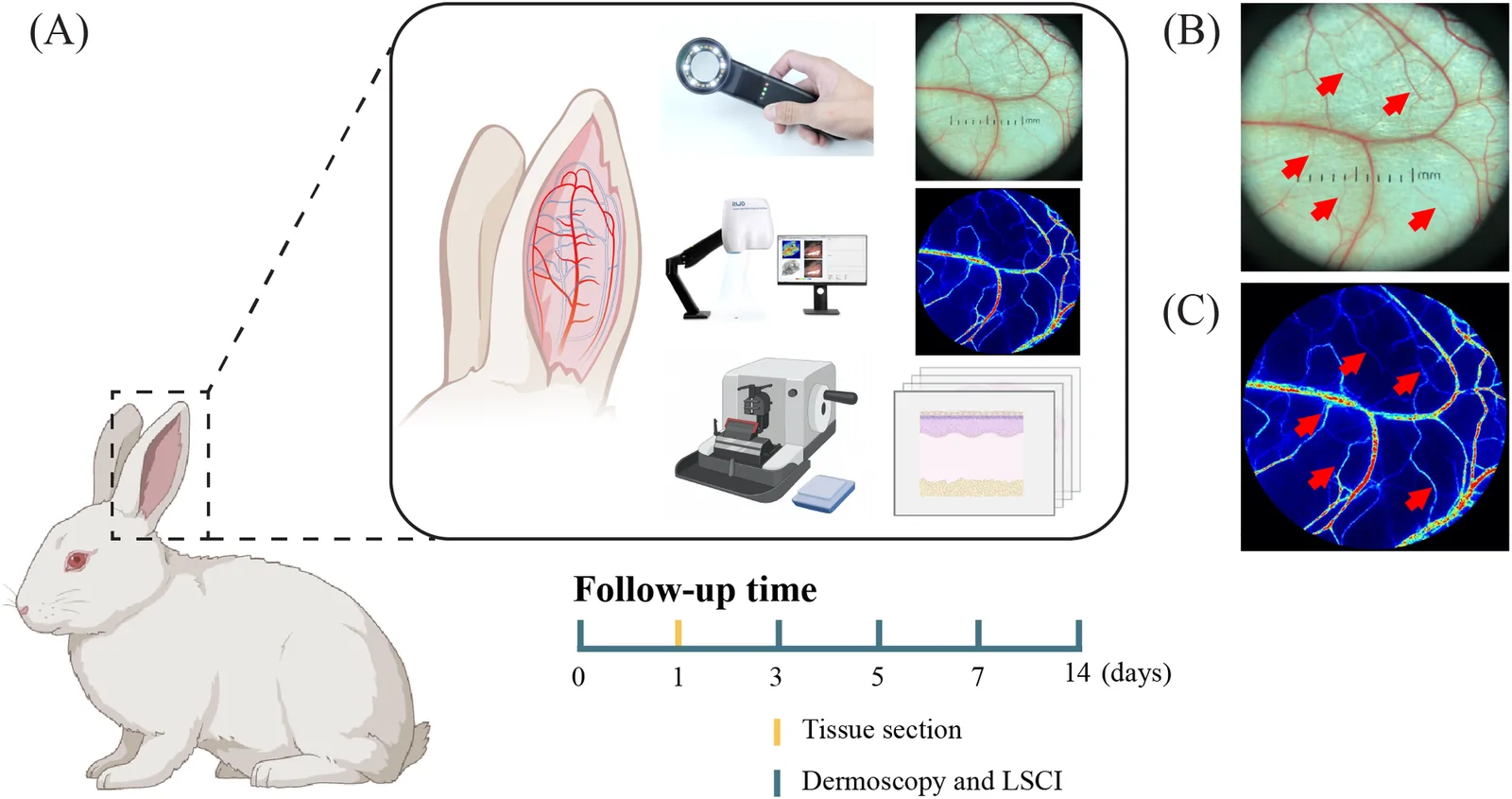
Schematic illustration of the evaluation modalities and timeline in a rabbit ear vascular model. **(A)** Following the establishment of the rabbit ear vascular model, vessels were longitudinally assessed at indicated time points using dermoscopy, laser speckle contrast imaging (LSCI), and histopathological analysis. **(B,C)** Schematic diagrams of vascular diameter and blood flow features acquired by dermoscopy and LSCI, respectively (red arrows indicate acquisition sites).

Follow-up time points included before treatment, immediately after treatment, 24 hours post-treatment, and on days 3, 5, 7, and 14 after treatment (Figures 1A,B).

**Figure 2:**
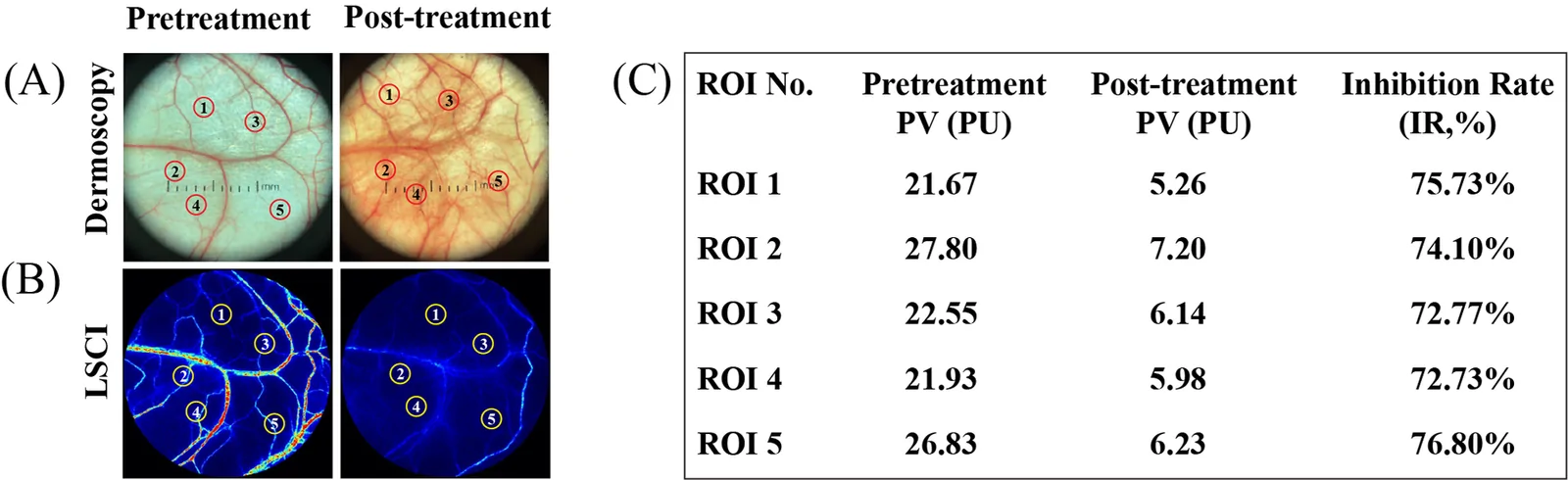
Representative workflow for ROI selection, multi-modal imaging, and quantitative perfusion analysis in the rabbit ear vascular model. **(A)** Representative dermoscopic images of the rabbit ear. Red circles (ROIs 1–5) indicate five branch blood vessels (BBVs; baseline diameter, 90–120 μm) selected as regions of interest (ROIs) for quantitative analysis. Only BBVs were included in the quantitative analysis to minimize variability associated with vessel diameter. **(B)** Corresponding laser speckle contrast imaging (LSCI) of the same five BBV ROIs identified in the dermoscopic images. The identical ROIs (ROIs 1–5) were used for perfusion measurements before and after treatment. **(C)** Quantitative summary of baseline and post-treatment perfusion values (PV, expressed in perfusion units [PU]) and calculated inhibition rates (IR, %) for ROIs 1–5. The inhibition rate was calculated as: IR (%) = [(PV baseline - PV post - intervention)/PV baseline] × 100%. Scale bar = 1 mm.

### 2.4 Histopathology

Histological sections were collected 24 h post-treatment and stained with H&E for vascular and inflammatory assessment. Histological evaluation focused on vascular injury, erythrocyte extravasation, inflammatory infiltration, and tissue edema, using a predefined semiquantitative scale (0 to +++++: 0, normal; +, minimal; ++, mild; +++, moderate; ++++, marked; +++++, severe/extensive). The same grading criteria were applied across all treatment groups. All sections were independently evaluated by two pathologists.

### 2.5 Statistical analysis

The image analysis software ImageJ was used for the quantitative analysis of blood vessels. The statistical software package GraphPad Prism 9 (GraphPad Software, Inc., La Jolla, CA, USA) was used for statistical analysis. Numerical data are presented as the mean ± standard deviation. The normality of the data was assessed using the Shapiro–Wilk test. For longitudinal comparisons of perfusion units (PU) and vascular inhibition rates (IR) across multiple fluence levels and time points, a two-way repeated-measures ANOVA was employed, followed by Tukey's *post-hoc* test for multiple comparisons between specific groups. Statistical significance is indicated as follows: ns, not significant; *p < 0.05; **p < 0.01; ***p < 0.001; ****p < 0.0001.

## 3 Results

### 3.1 Multimodal assessment of vascular responses in the rabbit ear model

The dorsal vasculature of the rabbit ear was used as an accessible model for investigating cutaneous vascular responses to laser treatment. A standardized multimodal assessment framework integrating dermoscopy, laser speckle contrast imaging (LSCI), and histopathology was established for longitudinal evaluation of vascular morphology, perfusion dynamics, and tissue responses (Figure 1). Dermoscopy clearly visualized the vascular distribution and morphology of the rabbit ear, revealing longitudinally oriented main blood vessels (MBVs) and fan-shaped branching patterns of smaller branch blood vessels (BBVs). The diameters of the MBVs and BBVs measured approximately 150–300 μm and 90–120 μm, respectively. LSCI was used for real-time monitoring of blood perfusion dynamics, with quantitative analysis primarily performed on BBVs within the 90–120 μm diameter range to assess changes in perfusion following treatment. Histological analysis was employed to evaluate vascular injury, erythrocyte extravasation, inflammatory cell infiltration, and edema, providing complementary assessment of treatment-related tissue responses. After PDL and IPL treatment, immediate vascular responses, including vasoconstriction, coagulation, vessel rupture, and erythrocyte extravasation, were observed, followed by varying degrees of vascular recovery over the 14-day follow-up period. Overall, the established multimodal framework enabled reproducible, longitudinal, and complementary assessment of vascular morphology, perfusion dynamics and tissue-level responses to PDL and IPL treatment.

### 3.2 Evaluation of PDL vascular responses using the rabbit ear vascular model

PDL is one of the most widely used vascular-targeted laser modalities. Two representative pulse durations (0.45 ms and 1.5 ms) were selected based on clinical practice. The calculated TRTs for BBVs (3.6–6.4 ms) and MBVs (10.0–40.2 ms) exceeded both pulse durations, thereby facilitating preferential thermal confinement within the target vessels during laser irradiation.

#### 3.2.1 Pulse duration of 0.45 ms

**Figure 3:**
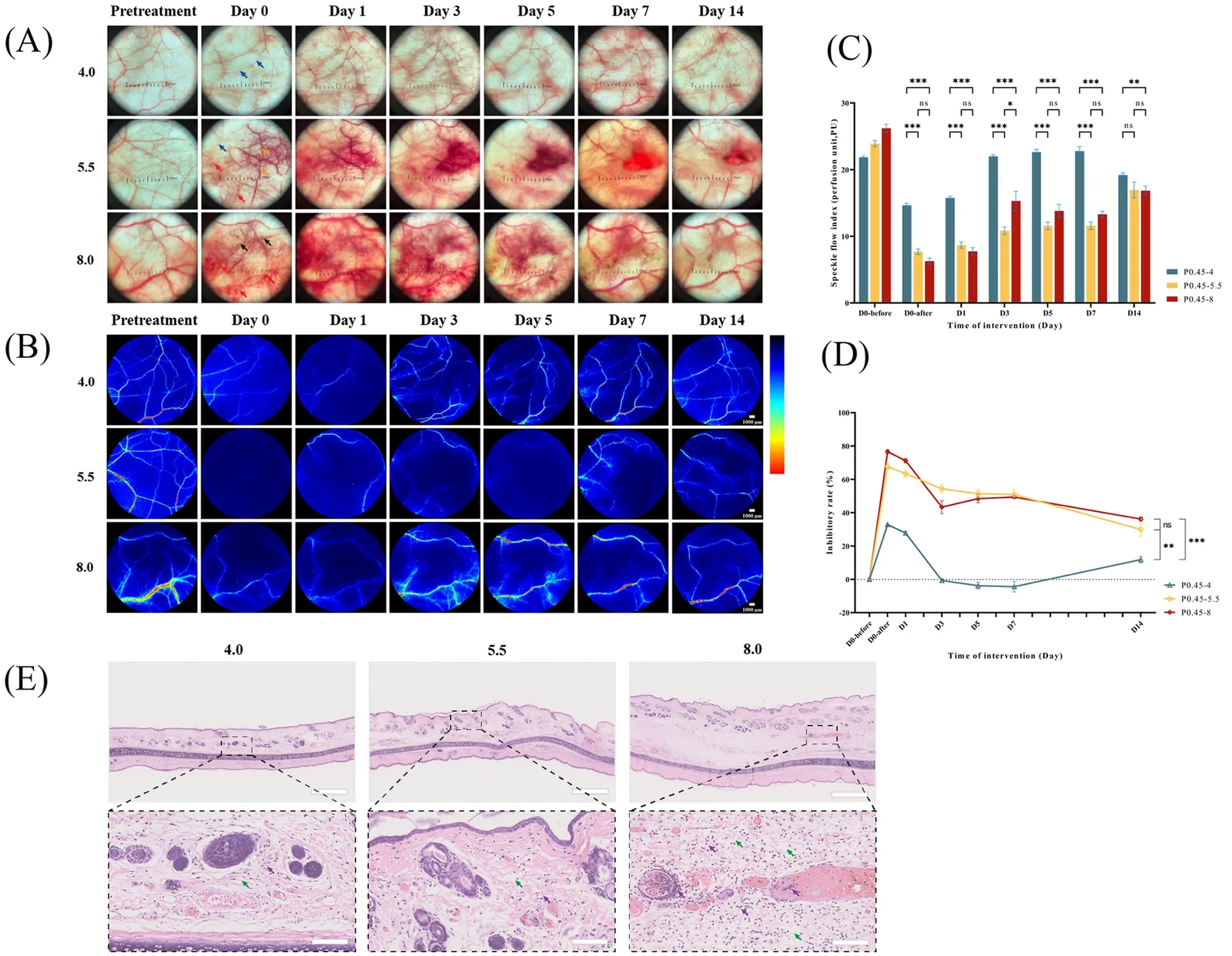
Longitudinal multimodal evaluation of vascular responses following PDL treatment with a 0.45 ms pulse duration. **(A)** Representative dermoscopic images of rabbit ear vasculature before treatment and at designated time points (Days 0–14) across three fluence groups (4.0, 5.5, and 8.0 J/cm^2^). Vascular constriction (blue arrow), vessel occlusion (black arrow), coagulation (orange arrow), and vessel rupture with erythrocyte extravasation (red arrow) are indicated. Scale bar = 1000 *μ*m. **(B)** Corresponding LSCI images of the same treatment regions. Scale bar = 1000 μm. **(C)** Quantitative analysis of vascular perfusion values (PV, perfusion units [PU]) measured by LSCI across the three experimental groups (25 vascular ROIs per fluence group). **(D)** Vascular inhibition rate (IR). Trends of the vascular inhibition rate during the 14-day follow-up period (25 vascular ROIs per fluence group). **(E)** Histopathological examination. H&E-stained cross-sections of rabbit ear tissue at 24 hours post-intervention. Inflammatory cell infiltration (purple arrow) and tissue edema (green arrows) are indicated. Top: 2.5×, scale bar = 1000 μm. Bottom: 100×, scale bar = 100 μm.

After laser irradiation at energy fluences of 4.0, 5.5, and 8.0 J/cm^2^ with a 0.45 ms pulse duration, dermoscopic observation revealed immediate vascular constriction, disappearance, coagulation, or rupture, accompanied by red blood cell leakage (Figure 3A). More pronounced morphological changes were observed at higher fluences in the representative images. LSCI showed an immediate loss of perfusion signals in the BBVs, whereas perfusion in the MBVs was only partially reduced. These findings suggest that a 0.45 ms pulse duration produced a marked acute reduction in perfusion, particularly in the smaller BBVs. By day 3, partial vascular recovery was evident, with LSCI perfusion signals reappearing earlier in MBVs than in the BBVs. By day 14, LSCI showed that some BBVs in the 4.0 J/cm^2^ group remained nonperfused, whereas perfusion in the MBVs had largely recovered in the 5.5 and 8.0 J/cm^2^ groups; most BBVs in these groups remained nonperfused (Figure 3B).

Consistent with these observations, the vascular inhibition rate (IR) increased markedly immediately after treatment and subsequently declined during follow-up, indicating progressive vascular reperfusion. The 4.0 J/cm^2^ group showed a greater reduction in IR over time, whereas the 5.5 and 8.0 J/cm^2^ groups retained higher IR values. At day 14, the IR values were 29.99 ± 21.46% and 36.06 ± 7.36% in the 5.5 and 8.0 J/cm^2^ groups, respectively; the difference between these two groups was not statistically significant (P = 0.18). Histological examination at 24 h after treatment provided complementary evidence of tissue responses. Severe inflammatory cell infiltration and tissue edema were observed in the 8.0 J/cm^2^ group (Figure 3E).

#### 3.2.2 Pulse duration of 1.5 ms

**Figure 4:**
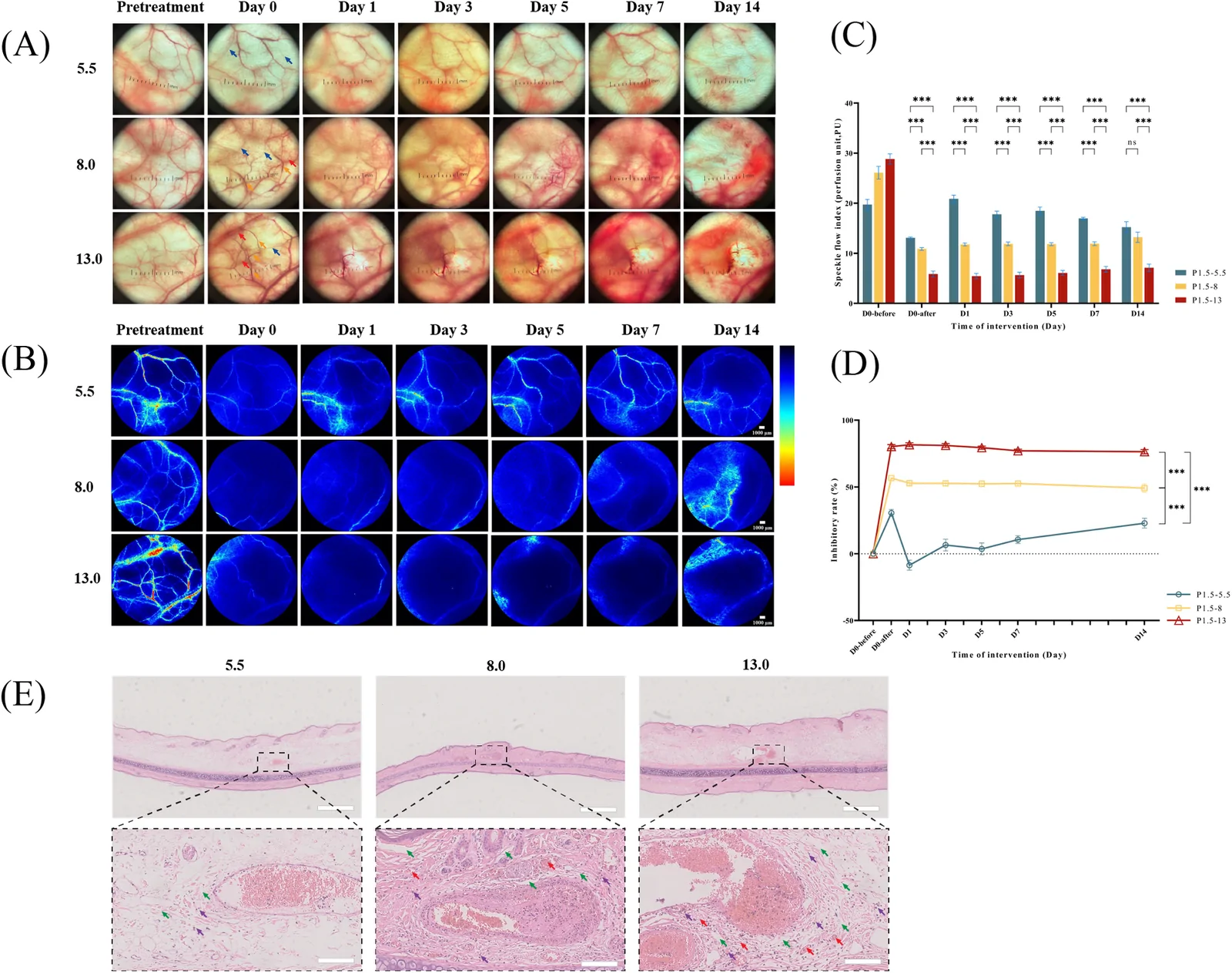
Longitudinal multimodal evaluation of vascular responses following PDL treatment with a 1.5 ms pulse duration. **(A)** Representative dermoscopic images of rabbit ear vasculature before treatment and at designated time points (Days 0–14) across three fluence groups (5.5, 8.0, and 13.0 J/cm^2^). Vascular constriction (blue arrow), coagulation (orange arrow), and vessel rupture with erythrocyte extravasation (red arrow) are indicated. Scale bar = 1000 μm. **(B)** Corresponding LSCI images of the same treatment regions. Scale bar = 1000 μm. **(C)** Quantitative analysis of vascular perfusion values (PV, perfusion units [PU]) measured by LSCI across the three experimental groups (25 vascular ROIs per fluence group). **(D)** Vascular inhibition rate (IR). Trends of the vascular inhibition rate during the 14-day follow-up period (25 vascular ROIs per fluence group). **(E)** Histopathological examination. H&E-stained cross-sections of rabbit ear tissue at 24 hours post-intervention. Inflammatory cell infiltration (purple arrow), erythrocyte extravasation (red arrow) and tissue edema (green arrows) are indicated. Top: 2.5×, scale bar = 1000 μm. Bottom: 100×, scale bar = 100 μm.

At a pulse duration of 1.5 ms, dermoscopic examination showed vascular constriction across all three fluence groups. In the 5.5 J/cm^2^ group, coagulative vascular changes accompanied by mild erythrocyte extravasation were observed (Figure 4A). LSCI revealed no significant change in blood perfusion immediately after treatment, with only a slight reduction observed by day 14 (Figure 4B). Histological analysis revealed intraluminal thrombus formation, with intact vessel walls, minimal perivascular exudation, and mild inflammatory cell infiltration in the surrounding tissues (Figure 4E).

At higher fluences (8.0 and 13.0 J/cm^2^), dermoscopic observations revealed immediate vessel coagulation, followed by progressive erythrocyte extravasation, which was most evident around day 7. During this period, the vascular borders became indistinct and “hazy” because of increased tissue edema and vascular wall disruption (Figure 4A). LSCI showed an absence of detectable perfusion signals in both MBVs and BBVs from immediately after treatment through day 7, a finding that is consistent with the macroscopic pallor in the treated area (Figure 4B). Histological analysis revealed dense erythrocyte clots within the lumen, features consistent with vascular occlusion in both the 8.0 and 13.0 J/cm^2^ groups. In the 13.0 J/cm^2^ group, vessel structures were disrupted and fragmented, accompanied by severe tissue edema, dermal matrix loosening, interstitial fluid accumulation, and dense inflammatory cell infiltration in the perivascular regions (Figure 4E).

### 3.3 Evaluating IPL vascular responses using a rabbit ear vascular model

In clinical IPL practice, filters that target hemoglobin absorption peaks, such as the “590 nm filter” and the “vascular filter”, are typically used to treat vascular skin disorders. Given the multiple adjustable parameters of IPL systems, this study employed a pulse duration of 5 ms, which exceeds the epidermal TRT while remaining below the vascular TRT, thereby facilitating preferential vascular heating while minimizing collateral thermal injury. In the double-pulse mode, a 30 ms inter-pulse delay was selected, which is longer than the epidermal TRT yet sufficiently short to preserve the cumulative thermal effect of consecutive pulses. These settings were adopted as the basis for subsequent experimental investigations.

#### 3.3.1 Single-pulse mode

**Figure 5:**
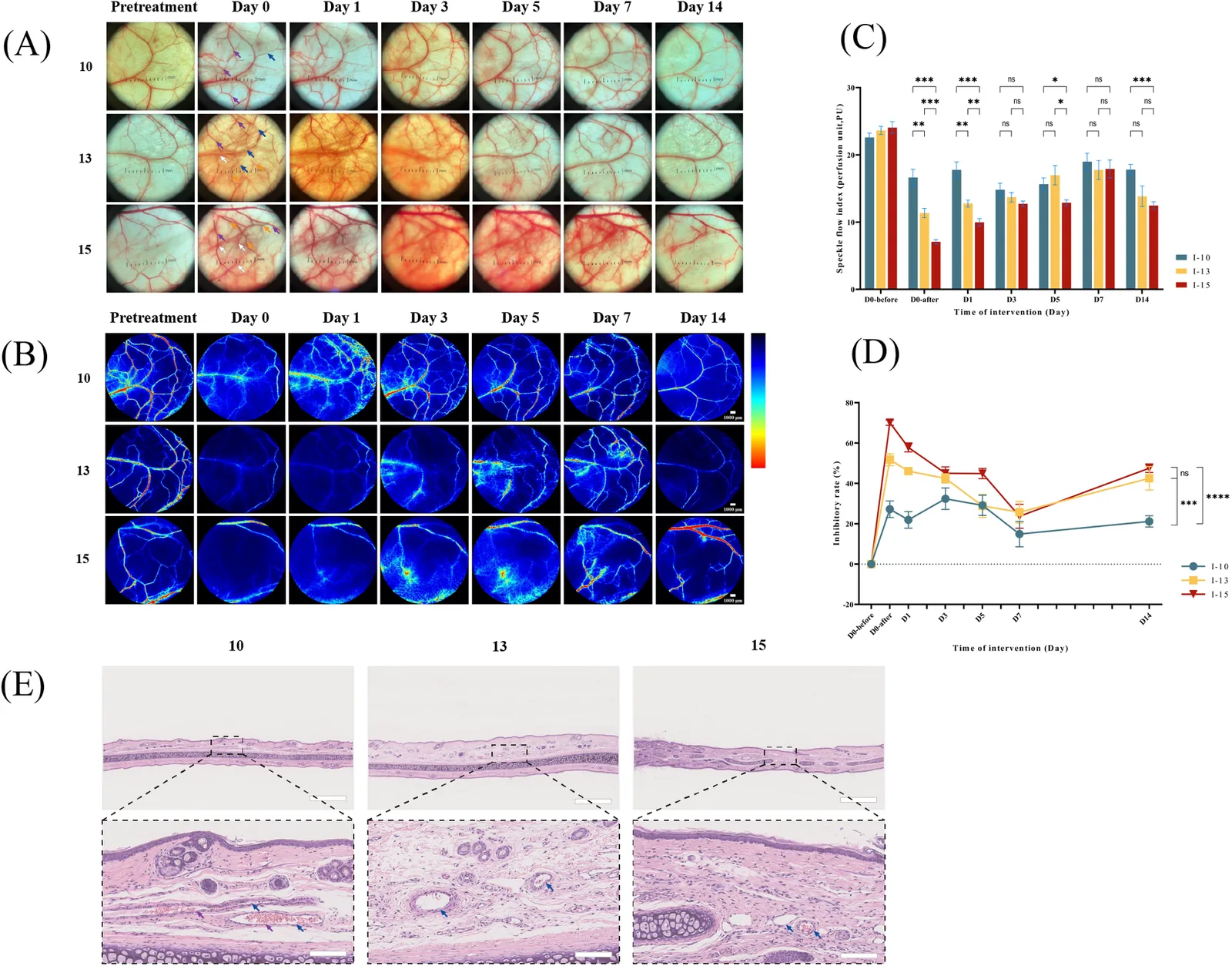
Longitudinal multimodal evaluation of vascular responses following IPL treatment in single-pulse mode. **(A)** Representative dermoscopic images of rabbit ear vasculature before treatment and at designated time points (Days 0–14) across three fluence groups (10, 13, and 15 J/cm^2^). Vasodilation (purple arrow), vascular constriction (blue arrow), coagulation (orange arrow), vessel rupture and exudation (white arrow) are indicated. Scale bar = 1000 μm. **(B)** Corresponding LSCI images of the same treatment regions. Scale bar = 1000 μm. **(C)** Quantitative analysis of vascular perfusion values (PV, perfusion units [PU]) measured by LSCI across the three experimental groups (25 vascular ROIs per fluence group). **(D)** Vascular inhibition rate (IR). Trends of the vascular inhibition rate during the 14-day follow-up period (25 vascular ROIs per fluence group). **(E)** Histopathological examination. H&E-stained cross-sections of rabbit ear tissue at 24 hours post-intervention. Dilated vascular lumen (purple arrow) and intact vessel wall (blue arrow) are indicated. Top: 2.5×, scale bar = 1000 μm. Bottom: 100×, scale bar = 100 μm.

Following single-pulse IPL irradiation, vessels in the 10 J/cm^2^ group showed early vasodilation, with histological analysis confirming lumen dilation, an intact vessel wall, and no erythrocyte extravasation. In contrast, at higher fluences of 13 J/cm^2^ and 15 J/cm^2^, vessels of different diameters, as well as different segments of the same vessel, exhibited heterogeneous responses, including dilation, coagulation, rupture, and erythrocyte extravasation. However, no intravascular thrombus formation was observed histologically (Figure 5E).

Immediately after treatment, LSCI measurements revealed a significant reduction in blood perfusion, followed by gradual recovery as vascular repair progressed. The perfusion inhibition rates for all the groups initially increased immediately after irradiation but subsequently decreased over time. The degree of perfusion inhibition was greater at higher fluences. (Figure 5D). Blood flow recovery was first observed around day 3, with the 10 J/cm^2^ group exhibiting rapid reperfusion and a less sustained inhibitory response. In the 13 J/cm^2^ and 15 J/cm^2^ groups, reperfusion was initially observed in the MBVs and subsequently extended to the BBVs, although a significant proportion of BBVs remained nonperfused (Figures 5B,C). Among the single-pulse settings, 15 J/cm^2^ showed the highest observed perfusion inhibition and more pronounced tissue responses. Dermoscopic imaging demonstrated persistent erythrocyte extravasation at day 7 and incomplete vascular recovery by day 14 (Figure 5A).

#### 3.3.2 Double-pulse mode

**Figure 6:**
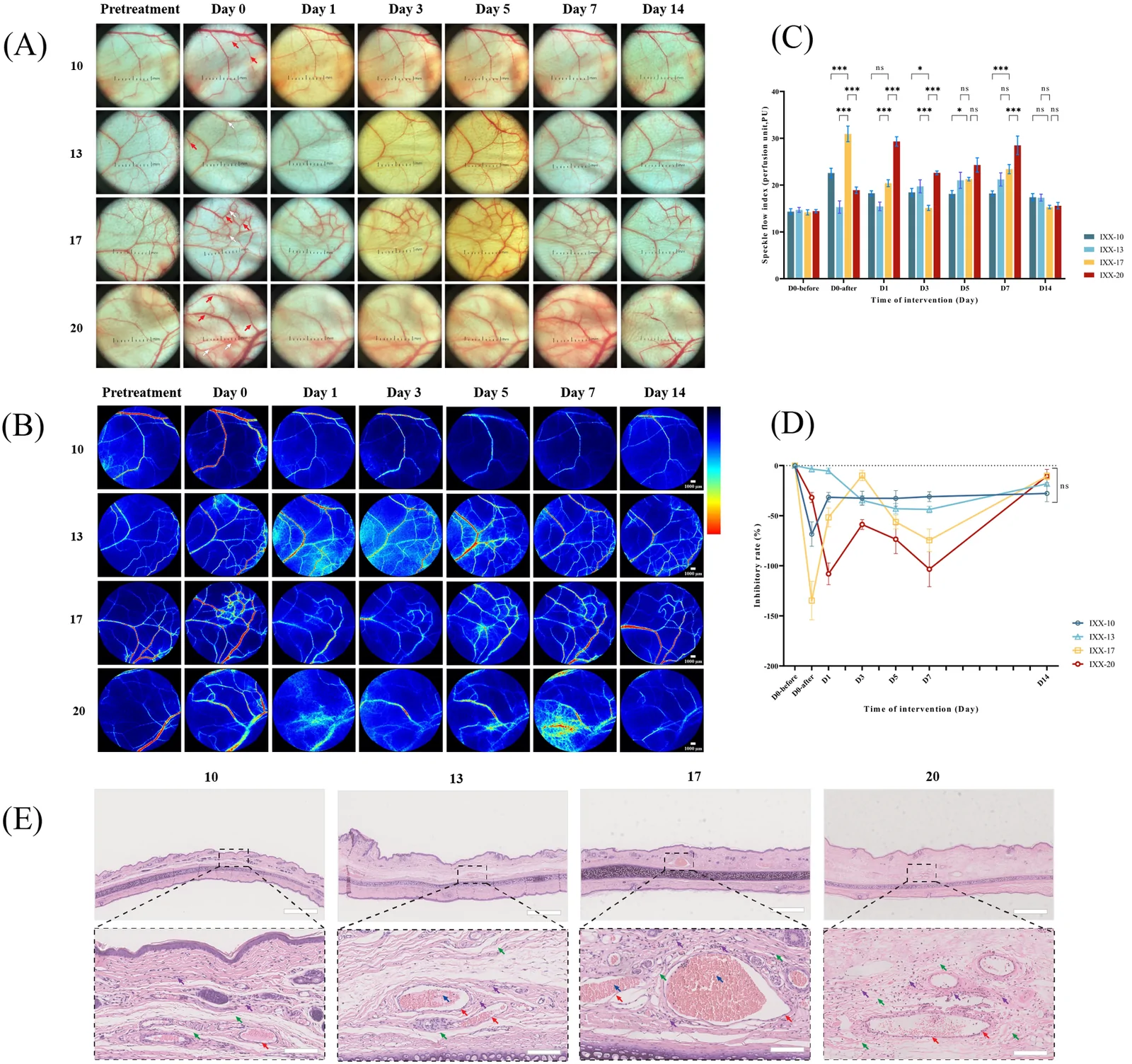
Longitudinal multimodal evaluation of vascular responses following IPL treatment with a double-pulse mode. **(A)** Representative dermoscopic images of rabbit ear vasculature before treatment and at designated time points (Days 0–14) across four fluence groups (10, 13, 17, and 20 J/cm^2^). Vasodilation (red arrow) and exudation (white arrow) are indicated. Scale bar = 1000 μm. **(B)** Corresponding LSCI images of the same treatment regions. Scale bar = 1000 μm. **(C)** Quantitative analysis of vascular perfusion values (PV, perfusion units [PU]) measured by LSCI across the four experimental groups (25 vascular ROIs per fluence group). **(D)** Vascular inhibition rate (IR). Trends of the vascular inhibition rate during the 14-day follow-up period (25 vascular ROIs per fluence group). **(E)** Histopathological examination. H&E-stained cross-sections of rabbit ear tissue at 24 hours post-intervention. Dilated blood vessels (red arrow), intraluminal thrombus (blue arrow), inflammatory cell infiltration (purple arrow) and tissue edema (green arrows) are indicated. Top: 2.5×, scale bar = 1000 μm. Bottom: 100×, scale bar = 100 μm.

After double-pulse IPL irradiation, vessels in all fluence groups showed immediate dilation accompanied by increased perfusion. LSCI revealed a transient increase in the blood perfusion rate, particularly in the 17 J/cm^2^ and 20 J/cm^2^ groups, followed by a gradual return toward baseline during follow-up (Figure 6C). Dermoscopic examination showed mild erythrocyte extravasation at later time points. Histological analysis at 24 hours confirmed vascular dilation and intraluminal thrombus formation. Greater perivascular inflammatory cell infiltration was observed at higher fluences (Figure 6E).

By day 14, the vascular morphology and perfusion levels had returned to baseline in all the groups, with no significant differences (*P* = 0.520) among the fluence groups. In the 20.0 J/cm^2^ group, a small number of smaller vessels were no longer detectable, but the overall perfusion rate remained unchanged. This may reflect compensatory perfusion within the remaining patent vessels.

## 4 Discussion

In this study, we applied an established rabbit ear vascular model to characterize laser-induced vascular responses and systematically evaluated the treatment parameters of pulsed dye laser (PDL) and intense pulsed light (IPL). A multimodal assessment incorporating dermoscopy, laser speckle contrast imaging (LSCI), and 24-hour histological analysis was employed to comprehensively evaluate vascular morphology, perfusion dynamics and tissue responses, thereby providing reliable experimental evidence for comparative vascular response assessment and parameter selection.

The rabbit ear vascular model offers favorable applicability and stability for translational research. The vascular anatomy of the rabbit ear shares similarities with that of human skin, in terms of layer structure, vessel distribution, and caliber (35). Human dermal vessels typically measure 10–150 μm in diameter, reaching 100–300 μm under pathological conditions such as scars or hemangiomas (36–38). Similarly, the rabbit ear harbors a dense dermal capillary network and main vessels (150–300 μm) situated between the dermis and perichondrium. Their branching pattern and range of vessel calibers provide a useful anatomical basis for investigating laser-induced responses (27, 28).

A major advantage of the rabbit ear model is the preservation of an intact skin barrier, thereby enabling longitudinal, noninvasive monitoring via dermoscopy and LSCI. In contrast, the mouse window chamber model requires invasive surgical implantation of a metal frame, with associated risks of anesthesia-related complications, infection, and tissue damage. Its vessels are thin, deeply situated, and poorly collateralized, which may result in localized ischemic necrosis (39). Moreover, this model limits the number of independent treatment sites per animal. The rabbit ear model circumvents these drawbacks by supporting multiple independent treatment sites per animal, thereby reducing inter-individual variability and the total number of animals required for parameter screening.

Consequently, the rabbit ear vascular model provides an accessible platform that reproduces selected features of human cutaneous vasculature, allowing real-time, noninvasive, longitudinal evaluation of vascular responses to laser therapy. Nevertheless, differences in epidermal thickness, melanin content, and vessel depth between rabbit ear and human skin may influence laser light attenuation and energy deposition; potential clinical translation should therefore consider anatomical site and skin phototype (35).

Distinct vascular responses were observed between PDL and IPL treatments because of their differing optical properties and mechanisms of action. PDL emits a vascular-targeted wavelength selectively absorbed by oxyhemoglobin, concentrating thermal energy within the vessel lumen and wall to primarily injure endothelial cells. Consequently, at lower fluences, damage is largely confined to intravascular and vessel wall structures with minimal collateral injury. As fluence increases, vessel disruption and extravasation become more severe and are accompanied by greater perivascular tissue damage and interstitial edema (40, 41). In contrast, IPL delivers nonselective broadband light absorbed by hemoglobin, melanin, and dermal water, generating heat both within and around vessels (42). At lower fluences, the modest temperature rise may induce paradoxical heat-mediated vasodilation, particularly under double-pulse configurations where the inter-pulse delay reduces peak heating rates; therefore, higher fluences are typically required to achieve meaningful vascular damage (43).

Owing to the inherent differences between PDLs and IPL, their treatment parameters are not directly comparable. Thus, in this study, the vascular responses of each modality at various fluences and pulse duration settings were independently evaluated to identify the specific parameters required for distinct vascular effects.

In the screening of PDL parameters, we found that a pulse duration of 0.45 ms effectively induced the occlusion of BBVs measuring 90–120 μm in diameter. Among the tested fluences, 5.5 J/cm^2^ produced the most consistent and complete occlusion. Under these conditions, MBVs also exhibited an immediate reduction in perfusion; however, reperfusion occurred rapidly. For MBVs measuring 150–300 μm in diameter, a pulse duration of 1.5 ms at 8.0 J/cm^2^ was associated with qualitative evidence of more sustained vascular occlusion during follow-up. Lower fluences were associated with incomplete coagulation and early reperfusion, whereas excessively high fluences produced more pronounced inflammatory and tissue responses. Overall, these findings highlight the importance of selecting pulse duration on the basis of the vascular diameter distribution of the target lesion. For smaller-caliber vessels, shorter pulse durations at lower fluences were associated with sufficient vascular closure and limited peripheral tissue responses. For larger vessels, qualitative observations suggested that longer pulse durations and higher fluences were associated with more sustained vascular occlusion and greater perivascular inflammatory responses.

For single-pulse IPL, 13.0 and 15.0 J/cm^2^ produced marked perfusion reductions in BBVs (90–120 μm), with no significant difference in inhibition rate between the two fluences. This finding is consistent with Moy et al., who reported that increasing light energy can promote vascular closure, but that further energy increases may provide limited additional benefit once substantial vascular inhibition has been achieved (43). Our results therefore suggest that, within the tested range, increasing fluence beyond 13.0 J/cm^2^ did not provide an additional perfusion-inhibitory benefit, indicating a possible response plateau rather than a simple dose-dependent relationship. In contrast, double-pulse IPL produced immediate vasodilation and increased perfusion, resulting in negative inhibition rates across the tested fluences. Similar vasodilatory responses have been reported following low-fluence, multi-pulse IPL (5-ms pulse duration, 30-ms delay) by Minh et al. in a mouse model (44). These findings suggest that vascular responses to IPL are influenced not only by fluence but also by pulse structure, highlighting the importance of energy delivery in parameter selection.

Overall, the findings show that while both PDLs and IPL are effective for treating cutaneous vasculature, they produce different vascular responses, indicating their suitability for distinct clinical approaches. This is particularly evident for IPL, for which variations in pulse mode, pulse duration, and fluence can induce markedly different vascular outcomes (45). Consequently, parameter selection should be based on the vascular characteristics and disease treatment plan for each patient. In the treatment of scars, where vessels range from 10–250 μm in diameter, a balance between efficacy and safety is crucial. PDLs or single-pulse IPL can effectively occlude small vessels while preserving larger trunks (46). Fluence should be kept moderate to prevent excessive thermal injury and inflammation, reducing the risk of hypertrophic scarring (47). For vascular lesions with larger vessels, such as hemangiomas and port-wine stains, where vessel diameters can reach several hundred micrometers, higher fluence and appropriately extended pulse durations are necessary to achieve effective photocoagulation (15, 23). Conversely, in ischemic, nonhealing wounds in which enhanced perfusion is desired, double-pulse IPL may serve as a potential strategy to promote vascular dilation and increase local blood flow, thereby supporting tissue repair and wound healing (44, 48). For all IPL treatments, fluence must be carefully selected to balance efficacy and potential side effects. Energy settings should also account for light-absorbing chromophores such as melanin and be adjusted to individual skin types (e.g., Fitzpatrick classification) (49).

This study has several limitations. First, the ranges of energy density and pulse duration were limited and did not cover longer pulse durations (e.g., 3.0 ms in PDL mode) required for treating large vascular lesions. Second, rabbit ear skin differs significantly from human skin in epidermal thickness, melanin content, vessel depth, tissue optical properties, and surrounding tissue composition; these factors should be considered in clinical translation. Third, this model represents normal physiological conditions, whereas clinical vascular lesions are often accompanied by epidermal hyperplasia and dermal fibrosis, which may further limit laser penetration depth; thus, the current findings may not fully reflect actual therapeutic efficacy under pathological conditions. Future studies should expand the parameter ranges and establish pathological models, such as those for scar or tumor transplantation, to enhance the clinical translatability of this multimodal assessment framework.

## 5 Conclusion

In this study, we innovatively applied the rabbit ear vascular model to evaluate laser treatment responses in cutaneous vascular diseases. The model offers several advantages, such as being noninvasive, stable, and repeatable, providing a reliable platform for therapeutic evaluation and long-term follow-up. Using this model, we evaluated key treatment parameters for PDLs and IPL. For PDL, a pulse duration of 0.45 ms at 5.5 J/cm^2^ produced effective closure of BBVs measuring 90–120 μm with limited peripheral tissue damage, whereas 1.5 ms at 8.0 J/cm^2^ was associated with qualitative evidence of more sustained occlusion of MBVs measuring 150–300 μm. For IPL, single-pulse treatment at 13.0 J/cm^2^ produced effective occlusion of 90–120 μm vessels, whereas double-pulse treatment primarily induced vasodilation. Overall, this study provides experimental evidence to guide laser parameter selection and model establishment for cutaneous vascular lesions. Future studies may expand this platform by incorporating pathological conditions and broader parameter settings, providing a stronger preclinical basis for the further evaluation and refinement of vascular laser treatment protocols.

## Data Availability

The original contributions presented in the study are included in the article/Supplementary Material, further inquiries can be directed to the corresponding authors.
