# Supplementary material for: Real-time vascular monitoring of PDLs and IPL for vascular treatment: effects of fluence and pulse duration on a rabbit ear model
Source: Front Med (Lausanne). 2026 Aug 28;13:1917018. doi: 10.3389/fmed.2026.1917018 (PMC13561808; doi:10.3389/fmed.2026.1917018)
Supplement: Supplementary file 1 [file Table1.docx]

Supplementary Material

# 1 Supplementary Figures and Tables

**Supplementary Table 1. Predefined operational criteria for dermoscopic assessment of vascular responses**

| **No.** | **Response** | **Operational definition** |
| --- | --- | --- |
| 1 | Vasoconstriction | Reduction in vessel caliber relative to baseline |
| 2 | Vasodilation | Increase in vessel caliber relative to baseline |
| 3 | Occlusion/disappearance | Loss or disappearance of the previously visible intraluminal blood column |
| 4 | Coagulative change | Persistent intravascular darkening or aggregated appearance |
| 5 | Vessel rupture | Discontinuity of the vessel wall with localized erythrocyte leakage |
| 6 | Erythrocyte extravasation | Visible erythrocyte accumulation outside the vascular lumen |

**Supplementary Table 2. Semi-quantitative summary of histopathological findings in rabbit ear vessels 24 h after laser and light treatment at different fluences.**

| **No.** | **Group** | **Device** | **Fluence** | **Pulse duration/ Mode** | **Vascular Injury** | **Erythrocyte Extravasation** | **Inflammatory Cell Infiltration** | **Oedema** |
| --- | --- | --- | --- | --- | --- | --- | --- | --- |
| 1 | P0.45-4 | PDL | 4 J/ cm^2^ | 0.45ms | ++ | + | + | ++ |
| 2 | P0.45-5.5 | PDL | 5.5 J/ cm^2^ | 0.45ms | +++ | ++ | + | ++ |
| 3 | P0.45-8 | PDL | 8 J/ cm^2^ | 0.45ms | +++++ | ++ | ++++ | ++++ |
| 4 | P1.5-5.5 | PDL | 5.5 J/ cm^2^ | 1.5ms | none | none | ++ | ++ |
| 5 | P1.5-8 | PDL | 8 J/ cm^2^ | 1.5ms | ++ | + | ++ | +++ |
| 6 | P1.5-13 | PDL | 13 J/ cm^2^ | 1.5ms | +++++ | ++ | ++++ | ++++ |
| 7 | I-10 | IPL | 10 J/ cm^2^ | 5ms, single pulse | none | none | none | none |
| 8 | I-13 | IPL | 13 J/ cm^2^ | 5ms, single pulse | none | none | none | none |
| 9 | I-15 | IPL | 15 J/ cm^2^ | 5ms, single pulse | none | none | none | none |
| 10 | IXX-10 | IPL | 10 J/ cm^2^ | 5-30-5ms, double pulsed | none | none | none | + |
| 11 | IXX-13 | IPL | 13 J/ cm^2^ | 5-30-5ms, double pulsed | none | none | + | + |
| 12 | IXX-17 | IPL | 17 J/ cm^2^ | 5-30-5ms, double pulsed | ++ | none | + | + |
| 13 | IXX-20 | IPL | 20 J/ cm^2^ | 5-30-5ms, double pulsed | +++ | + | ++ | ++ |
